# Relaxing life of the city? Allostatic load in yellow-bellied marmots along a rural–urban continuum

**DOI:** 10.1093/conphys/coy070

**Published:** 2018-12-20

**Authors:** Kirsten Price, Charles Kittridge, Zach Damby, Stephen G Hayes, Elizabeth A Addis

**Affiliations:** 1 Biology Department, Gonzaga University, AD-5, 502 E Boone Ave, Spokane, WA, USA; 2 College of Veterinary Medicine, Washington State University, Pullman, WA, USA

## Abstract

Urban environments are expanding. As rural areas are urbanized, animals living in those environments must respond. Examinations of ecological responses to urbanization are abundant, but much less work has focused on the physiological responses driving those ecological patterns, particularly in mammals. Whether an animal interprets urbanized environments as stressful or not can help us understand, and even predict, the likelihood of individuals persisting in urbanized areas. Unpredictable events can cause stress and responses to such events can deplete limited stores of energy. Differences between required and available energy is termed allostatic load and is an indicator of stress. Allostatic load, and hence stress, is correlated with baseline levels of the metabolic hormones, glucocorticoids. We examined allostatic load in yellow-bellied marmots along a rural–urban gradient through analysis of fecal glucocorticoid metabolites (FGMs). We used GIS data and ‘on-the-ground’ measurements to quantify the degree of urbanization. We collected fecal samples from males and females of all age classes at six sites along this continuum. Female marmots had higher FGMs than males. All age groups of marmots exhibited a parabolic relationship between the degree of urbanization and FGM levels. In general, adult marmots had higher FGMs in more rural than urban environments, and both juveniles and yearlings had exhibited higher FGM levels in more urban environments.

## Teaser

We measured fecal glucocorticoid metabolites (FGMs), an indicator of an animal’s response to stress, in yellow-bellied marmots along a rural–urban continuum. Our findings suggest that adult marmots are less stressed in urban environments compared to rural ones, while young are more stressed in rural environments than urban ones.

## Introduction

With each passing year, urban expansion continues: rural to urban landscape conversion is incessant (Grimm *et al.*, 2008; Hahs *et al.*, 2009; Seto *et al.*, 2011). Urbanization is frequently reported as a leading cause of local extinction because of such factors as habitat loss, degradation of remaining habitat and increased competition from non-native species (McKinney, 2008). Therefore, organisms that live in such zones must adapt, acclimatize or move to different habitats in order to avoid death (McKinney, 2002; Shochat *et al.*, 2006). A great deal of work has focused on ecological patterns of animals that do well in urban environments and those that do not (e.g. Blair, 2004; Liker *et al.*, 2008). However, much less is known about the physiological mechanisms that allow animals to survive, or not survive, in urban environments. An animal’s physiological response to an environment reveals how an animal perceives its environment. This information is useful, particularly when dealing with conservation efforts, because physiological changes occur prior to population-level changes (Madliger and Love, 2014).

Numerous parameters can be measured to assess an animal’s physiological condition, but one key factor is an animal’s response to stressful events or a stressful environment. When dealing with an urban landscape, the pertinent question is if animals interpret the environment as stressful. Because chronic stress is deleterious (e.g. Romero *et al.*, 2009; Juster *et al.*, 2010), if animals find urban environments stressful, they are much less likely to survive and reproduce in them. Such animals are called ‘urban avoiders’ (McKinney, 2002) because they either leave or avoid urbanized areas. However, if animals do not find urban environments stressful, they are much more likely to survive and reproduce in these habitats. Such animals that use both naturally available resources and human subsidies can be termed ‘urban adaptors’. For example, woodchucks (*Marmota monax*) have been classified as urban adaptors because even though they are found in rural areas, they are also found in great abundance in urbanized zones (Lehrer and Schooley, 2010). Those that rely almost entirely on human subsidies, such as house sparrows (*Passer domesticus*), can be termed ‘urban exploiters’ (McKinney, 2002).

Recent works (Bonier, 2012; Fokidis *et al.*, 2009; Foltz *et al.*, 2015; Grunst *et al.*, 2014; Lyons *et al.*, 2017; Partecke *et al.*, 2006; Wright and Fokidis, 2016) have shown that endocrine traits, particularly those associated with the hypothalamo–pituitary–adrenal (HPA) axis, can facilitate acclimatization to urban areas. The HPA axis is integral to regulating both an organism’s energetic demands, and, hence its responses to stressful situations, through the regulation of glucocorticoid production in the adrenal gland. Energetic demands fluctuate based upon predictable variables such as time of year and day (Busch and Hayward, 2009). However, unpredictable events, such as a predation encounter or a shortage of food, can lead to increases in glucocorticoid levels, because, amongst several reasons, responses to such events require more energy. Specifically, the difference between required and available energy is termed allostatic load (Busch and Hayward, 2009; McEwen and Wingfield, 2003). More generally, allostatic load is the cumulative stress and physiological wear and tear that negatively affects animals’ functioning. Therefore, when allostatic load increases, particularly when energetic demands surpass energy available, frequently so do baseline levels of glucocorticoids (Bonier, 2012; Busch and Hayward, 2009; Landys *et al.*, 2006). Events that cause such increases can be called stressors. Measuring baseline glucocorticoid levels can serve as an approximate measure of an animal’s allostatic load, and, therefore, can be used as an indicator of stress. Glucocorticoid levels are only an approximate measure of allostatic load because other factors can also affect those circulating levels.

The vast majority of studies examining the effects of urbanization on glucocorticoid levels have been conducted in reptiles, specifically birds and lizards (e.g. Bonier *et al.*, 2007; French *et al.*, 2008; Liker *et al.*, 2008; Meillère *et al.*, 2015; Angelier *et al*., 2016), with much less work focusing on free-living mammals (Reeder and Kramer, 2005, but see Lyons *et al.,* 2017).

In this study, we examine the effects of an urban environment, in contrast to a rural one, on allostatic load, measured through levels of fecal glucocorticoid metabolites (FGMs), in a mammal, the yellow-bellied marmot (*Marmota flaviventris*). As we are interested in the animals’ cumulative response to the environment, it is important to measure integrative levels of glucocorticoids rather than brief ‘snapshots’ (Dantzer *et al.*, 2014; Madliger and Love, 2014). FGMs provide a non-invasive method to measure these integrative levels of glucocorticoids. Fecal levels of glucocorticoid metabolites are a result of the accumulation of glucocorticoids released over the period of time feces were produced.

We chose to study the yellow-bellied marmot both because marmots are found in abundance in urban and rural environments and because resources and predation vary between these two environments. Studying the physiology of an animal that at the very least maintains population levels in an urban environment can provide us with insight about how such an animal is able to succeed in such an environment. From these results, we can make predictions about physiological responses to urban environments of animals with lower population densities, particularly those of conservation concern. We hypothesize that allostatic load will vary between urban and rural populations in yellow-bellied marmots. More specifically, as we are using FGMs as indicators of allostatic load, we predict that FGMs will vary between urban and rural populations.

## Methods

### Rural–urban continuum

Because landscapes are highly variable, classifying sites as simply ‘urban’ or ‘rural’ is artificial. Therefore, using both ‘on-the-ground’ and GIS data, we constructed a rural–urban continuum from which we could extract urbanization scores for each field site. The construction of this continuum allowed us to assess the effect of urban development as a continuous variable. Other studies have constructed similar continuums (Liker *et al*., 2008; Lehrer *et al.*, 2012, 2010; Foltz *et al*., 2015; Meillère *et al.*, 2015), but most studies use a more arbitrary approach of assigning sites as ‘urban or rural’ (Partecke *et al*., 2006; French *et al.*, 2008; Mccleery, 2009; Ordeñana *et al*., 2010; Davies *et al*., 2013; Moller *et al.*, 2013; Nelson *et al.*, 2015).

At each field site, we collected ‘on the ground’ data of the number of people, dogs, cars and bikes that passed by in an hour. We collected these data between 7:00 and 13:00 for 3 days at all sites. Values included in the construction of the continuum were averages of those 3 days. We additionally measured sound intensity at each location. Sound intensity was measured by an American Recorder Technologies SPL-8810 sound meter set to HiDBC. Sound intensity was measured hourly between 7:00 and 13:00 for 3 days at all sites. Like the pedestrian traffic data, the sound intensity measurements were averaged for those 3 days.

We determined the type of land cover at a given trapping site using National Land Cover Database 2011 data with a 100-m-diameter buffer zone around trap sites (Homer *et al.*, 2015). From the National Land Cover Database, we were able to extract numerical values of both intensity of development and habitat type for each trapping location based upon the percent of the 100-m-diameter buffer zone that fell into specific development and habitat categories. Specifically, we extracted values for each of the following categories: developed-open space (impervious surfaces accounting for <20% of total cover), developed-low intensity (impervious surfaces account for 20–49% of total cover), developed-medium intensity (impervious surfaces account for 50–79% of the total cover), developed-high intensity (impervious surfaces account for 80–100% of the total cover), evergreen forest (areas dominated by trees generally >5 m tall, and >20% of total vegetation cover, and more than 75% of the tree species maintain their leaves all year), shrub/scrub (areas dominated by shrubs; <5 m tall with shrub canopy typically >20% of total vegetation; includes true shrubs and young trees in an early successional stage), or grassland herbaceous (areas dominated by graminoid or herbaceous vegetation, generally >80% of total vegetation). Generally, the greater the intensity of the development, the less area is devoted to natural habitats (like evergreen forest, shrub/scrub and grassland).

We used a principal component analysis to combine the ‘on-the-ground’ data with the GIS information into one variable for which we could extract a rural–urban score (RUS) for each site. While we had an abundance of environmental variables (nine in total, as described above), we only wanted to include those that contributed significantly to explaining the variance between sites. We iteratively determined which variables had an effect. More specifically, we tried all combinations of variables until we found a combination that explained the most variance.

### Study species and sites

The yellow-bellied marmot (*M. flaviventris*) is a medium-sized sciurid rodent found in western North America from Colorado to southern Canada (Frase and Hoffmann, 1980). In the southern area of their range, they are found up to 3000 m in elevation (Armitage, 2014) but in northern parts (e.g. Eastern Washington) they are found as low as 300 m (pers. obs.). Depending upon altitude, marmots will begin hibernation in as early as August and emerge as early as March. Copulation occurs in the first several weeks after emergence from hibernation. Gestation is approximately 4 weeks long (Frase and Hoffmann, 1980). Yellow-bellied marmots are polygynous cooperative breeders, with one male having a harem of several females (Armitage, 1962). The subspecies *M. flaviventris avara* found in the greater Spokane area (Frase and Hoffmann, 1980) is unique because it is one of the few subspecies that is found in an urban environment.

**Figure 1:**
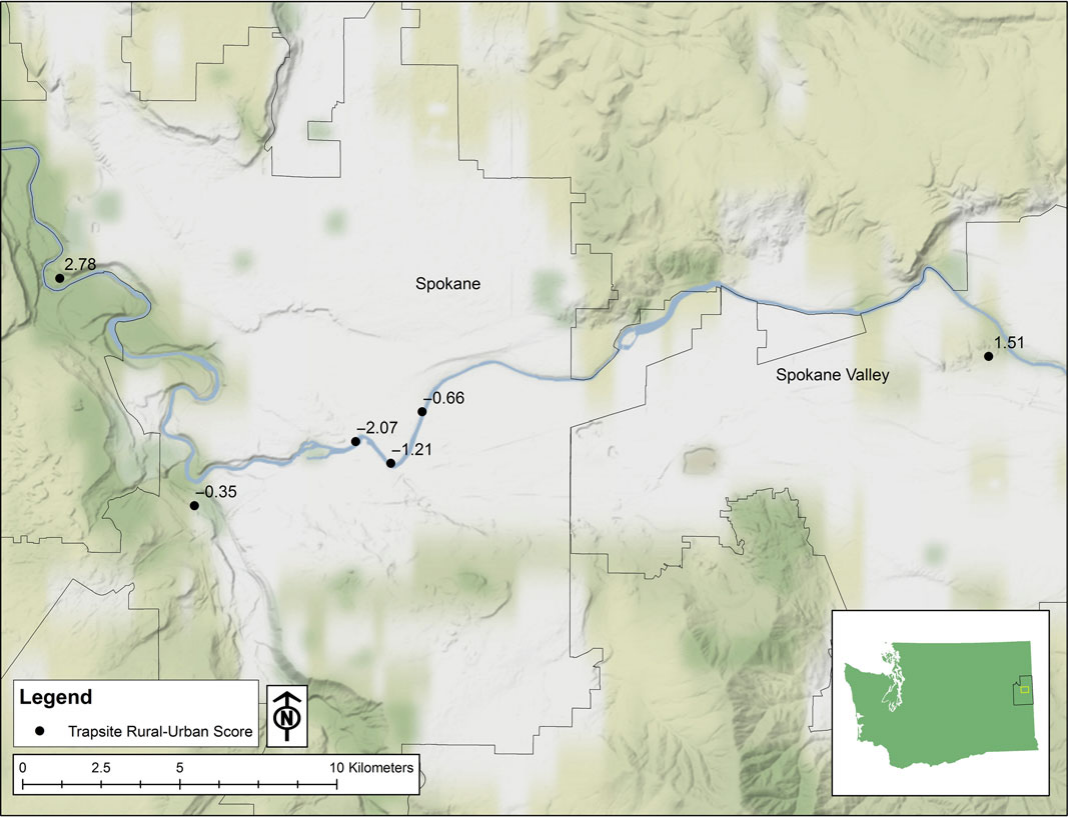
Map of the trapping sites (six in total) for yellow-bellied marmots in the Spokane, WA area. Values refer to the RUS for each field site. The map was created in ArcGIS ArcMap v10.1 (Environmental Systems Research Institute, 2012).

We trapped marmots at six locations in and around Spokane, Washington (Fig. 1; SOM Table 1). Between May and July of 2014–16, we collected samples from 97 live trapped-individuals. By May, mating and parturition have already occurred. Females were distinguished from males by either the presence of enlarged nipples or anogenital distance. All but one adult female caught had enlarged nipples; therefore, we could not investigate differences in reproductive substate. Individuals were also categorized by age based upon mass (Armitage, 2014): juvenile (first year of life), yearling (second year of life) and adult (older than 2 years). For individuals who were caught more than once, we removed all but the initial samples because of the very limited sample size of repeat captures (only 6 of the 97 were caught more than once). See SOM Table 2 for samples’ sizes of each age and sex group for each field site. All individuals were caught between 7:00 and 13:00. Traps were baited with apples, dandelions and ramen and were checked hourly. Upon capture, the marmot was weighed and a unique ear tag was applied for future identification. Feces were collected from the trap upon release of the marmot. All works were approved by the Gonzaga University IACUC. Annual permits were acquired from the Washington Department of Fish and Game.

**Table 1:** Contributions of each variable to principle component 1 for the PCA

Variable	PC 1 loadings
Developed, low intensity	−0.45
Developed, medium intensity	−0.32
People/bikes/dogs/cars	−0.30
Evergreen forest	0.49
Shrub/scrub	0.43
Grassland, herbaceous	0.42
Percent of variance explained	54.3

Principle component 1 was used to determine the RUS for each field site. This list includes all variables included in the PCA. More positive values refer to more rural sites.

**Table 2:** Coefficient estimates and significance tests for the final model, which predicts the effect of sex, age and RUS on fecal glucocorticoid metabolites (log FGM)

Model effect	Estimate (SE)	*P*-value
Intercept	6.712 (0.069)	<0.01
Sex (Male)	−0.177 (0.075)	0.02
Age (Yearling)	−0.044 (0.105)	0.68
Age (Juvenile)	0.156 (0.087)	0.07
RUS	0.138 (0.043)	<0.01
RUS^2^	−0.057 (0.023)	0.02
Age (Yearling) * RUS	−0.203 (0.076)	<0.01
Age (Juvenile) * RUS	−0.192 (0.062)	<0.01

Our model predicts that FGM is greatest in female juveniles, followed by adult females. The significant interaction provides evidence that adults experience their greatest FGM response at higher RUS values than juvenile or yearling age classes.

### Fecal extraction and enzyme-linked immunosorbent assay

Upon collection, feces were frozen at −20°C. Within 12 months of collection, feces were freeze-dried and hormones were extracted. For extraction of hormones, we used the pulse-vortexer protocol described by Wasser *et al.* (2010). Briefly, feces were first freeze-dried to allow for comparisons to be made based upon dry weight. Second, corticosterone metabolites were extracted using 70% ethanol and pulse vortexing followed by centrifugation. Hormone extracts were stored at −20°C until measurement.

**Figure 2:**
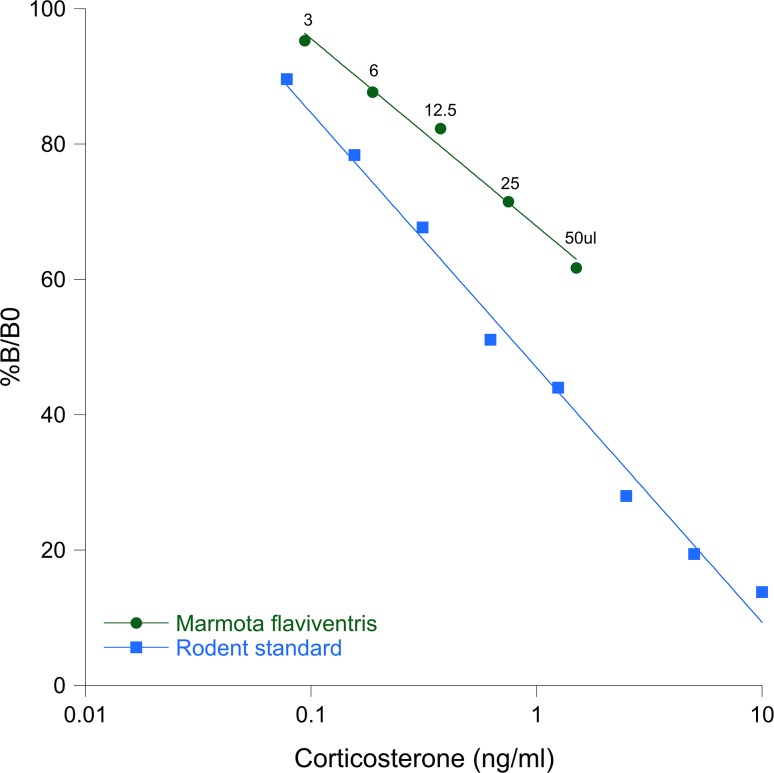
Validation of the rodent EIA for corticosterone in *M. flaviventris*. This figure shows the rodent standards of known concentration are parallel to a serial dilution (volumes of pooled plasma above curve, with 50μl representing the initial undiluted pool) for *M. flaviventris*. %*B*/*B*_0_ is the percent of maximal binding, with *B* being the amount of binding of the sample and *B*_0_ being the maximum binding possible in the assay.

We measured FGM levels using an enzyme-linked immunosorbent assay (EIA; Arbor Assays cat# K014). We validated this assay for *M. flaviventris* by comparing a pooled fecal extract serial dilution to the standard curve supplied with the kit (5000–78.125 pg/ml). We created a fecal extract pool (a combination of equal volumes of fecal extract from five individuals). Starting with 50μl of the pool, we serially diluted it to create a curve of the following dilution factors: undiluted, 1:2, 1:8; 1:64, 1:1024. Both the standard and the fecal extract pool curves were run in duplicate. The percent binding (*B*/*B*_0_) of the marmot and assay standard curves are shown in Fig. 2, showing the two curves are parallel, confirming the acceptable usage of the rodent Arbor Assay EIA corticosterone kit for hormone measurement. All samples were assayed in duplicate in a single assay. An identical plasma pool was run in all plates to determine intra-assay variation. Intra-assay variation was 4.14%.

### Statistical analysis

All FGM data were log transformed to meet normality assumptions. We first determined if marmot mass, year and/or date (expressed as Julian date) affected FGM levels using linear regressions. As none of these variables had a significant effect, they were excluded from our final analysis. Based upon graphical analysis of our data, we concluded that there was a quadratic effect of RUS on FGM levels. Therefore, we added the polynomial term of RUS^2^ to our linear model, which also included RUS and the factors of sex, age and all of the associated two- and three-way interaction effects. We used Akaike Information Criterion (AICc with a bias correction for small sample sizes; Burnham and Anderson, 2003) to identify the most parsimonious model with the greatest explanatory power. Our final model with the lowest AICc value was as follows:

$$Y\;=\;\mu +\mathrm{Sex}+\mathrm{Age}+\mathrm{RUS}+{\mathrm{RUS}}^{2}+(\mathrm{RUS}\;*\;\mathrm{Age})+\varepsilon$$

where *Y* is the dependent variable of log (FGM), μ is the grand mean and ε is the error term. We used model-adjusted means to compare effects of observed levels of RUS on FGM. Data were analyzed in JMP Pro v.13.0 (SAS Institute Inc., 2016) and R (R Development Core Team, 2018). We used R to fit and evaluate regression models, estimate adjusted means and generate model predictions for graphical interpretations. We used contrasts to identify significant differences among age groups within observed levels of RUS, with Tukey-corrected *P*-values for the family of three age-group estimates (Lenth, 2016).

## Results

### Rural–urban score

**Figure 3:**
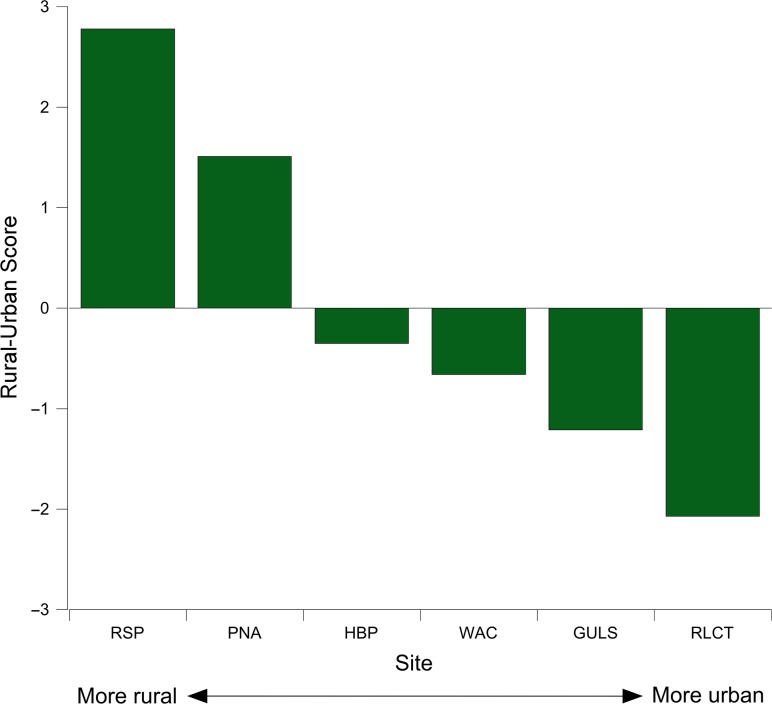
The RUS of each field site. Note that the higher the score, the more rural the field site. RUS scores are values of PC 1.

The principal component (PC) 1 explained 54.3% of the variance observed at study sites from the ‘on-the-ground’ and GIS data (Table 1; mean scores for each variable are given in SOM Table 3). The complete list of variables included in the PCA are also shown in Table 1. The PC 1 value for each site became that site’s RUS (Fig. 3). Generally, areas with more vegetation and less development resulted in a higher score; the higher the score, the more rural the site. The variables of noise, developed, open space and developed, high intensity, had eigenvalues of <0.05 and so were excluded from the analysis.

### Fecal glucocorticoid metabolites

Because this study included samples collected over 4 years, we tested if year of capture had an effect on FGM levels. Year did not (*F*_1,141_ = 0.36; *P* = 0.55). Further, because we collected samples over a 2-month time span, we tested if date of capture had an effect on FGM levels. Date of capture did not affect FGM levels (*F*_1,139_ = 0.23; *P* = 0.63). The mass of the marmot also had no effect on FGMs (*F*_1,100_ = 0.10; *P* = 0.75).

**Figure 4:**
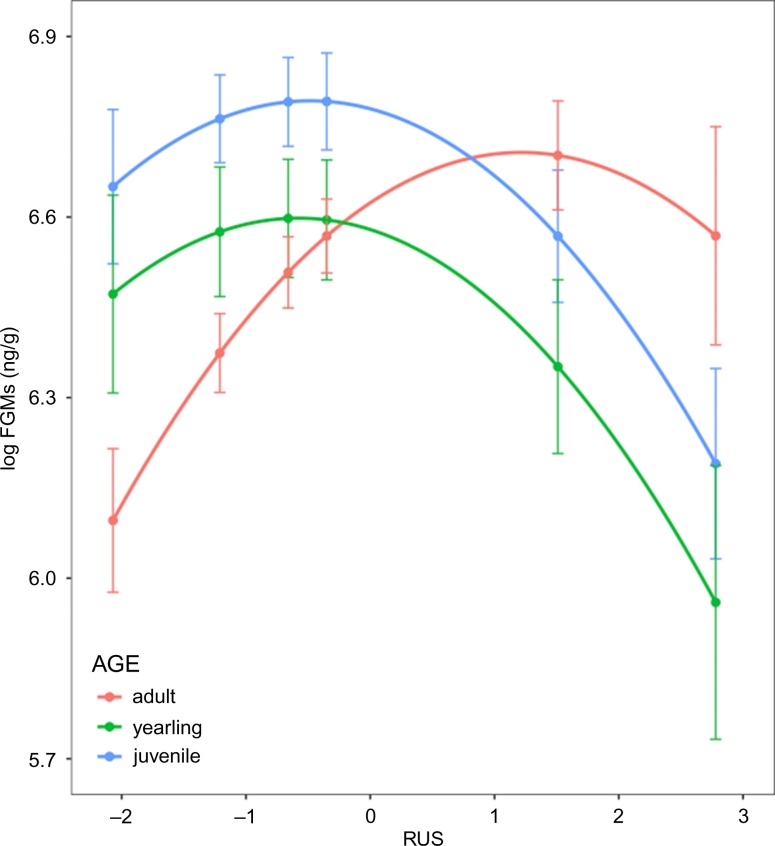
The parabolic relationship between RUS and FGMs. Note that the limits of the *y*-axis extend from 5.7–6.9 ng/g. There is a significant interaction between age and RUS on log FGM levels. The vertices of the curve differ between adults and young. Adults have higher log FGM levels in rural locales than young marmots, but young marmots have higher log FGMs in urban sites. Plotted points are model-adjusted means with standard error bars.

Female marmots had FGMs that were on average 1.194 ng/g greater than male marmots (*P* = 0.020; Table 2). RUS had a significant negative quadratic effect on log FGM (as determined by the inclusion of RUS^2^ in the model; *P* = 0.02), producing the greatest predicted log FGMs at intermediate RUS values. In addition, RUS interacted with each level of age (adult, yearling, juvenile), significantly shifting the vertices of each parabolic response (*P* < 0.01; Fig. 4). The vertex of the adult curve is shifted right compared to those of the young (juveniles and yearlings). Contrasts among age groups within observed levels of RUS reveal the effect of the interaction in the model. Adults had significantly lower FGM than youths at RUS values <−0.35. At an RUS of 2.78, the highest level we observed, adults had a significantly greater FGM than yearlings (Fig. 4).

## Discussion

Ecological studies on animal patterns associated with urbanization have been extensive, but physiological studies are less common, particularly in mammals. In this study, we investigated if the degree of urbanization of an environment affects marmots’ allostatic load, as measured through FGMs. We found that females had consistently higher FGM levels than males. Additionally, we found that the relationship between FGMs and urbanization is parabolic. Finally, there is an interaction between urbanization and age, resulting in different peaks of FGMs along the urban–rural continuum between adults and young.

### Sex effects

We found that females had significantly higher FGMs than males, regardless of age. Studies in other species have shown equivocal results of females or males having higher glucocorticoid levels. For example, male Syrian hamsters (*Mesocricetus auratus*; Chelini *et al.*, 2010) and male American pikas (*Ochotona princeps*; Wilkening *et al.*, 2013) had higher levels than females, but female common voles (*Microtus arvalis*; Navarro-Castilla *et al.*, 2014), prairie voles (*Microtus orchogastor*; Taymans *et al.*, 1997) and red-backed voles (*Clethrionomys gappei*; Kramer and Sothern, 2001) had higher FGM levels than males. This seems to be a common trend in many mammalian species (Reeder and Kramer, 2005).

However, this finding is contrary to previous work in captive and wild-caught yellow-bellied marmots in Colorado (Smith *et al.*, 2012). This contradiction is surprising, as one would expect to find the same pattern in sex differences within a species. What could account for these differences? No single explanation is obvious. The reproductive state of females has been shown to affect FGM levels in yellow-bellied marmots (Smith *et al.*, 2012). Smith *et al.* (2012) found that pregnant females had higher FGMs than lactating females. However, considering that at our field sites the marmots breed in March, we find it unlikely that the females in our study population were pregnant as we began collecting samples in late May and continued through Mid-July. Regardless, Smith *et al.* (2012) found that pregnant female FGM levels were still lower than those of males, suggesting that another reason might account for the disparity between our results and those from Colorado. Other possible, but highly speculative, explanations for the contradictory findings between males and females in Colorado and Washington could be differences in metabolic demands (Touma *et al*., 2003; Goymann, 2005; Busch and Hayward, 2009), variances in social dynamics because of differing environments (Creel *et al.*, 2013), or seasonal timing of sample collection, with samples from the wild populations in Colorado collected for a longer period each season than in this study (Smith *et al.*, 2012).

### Parabolic relationship between RUS and FGMs

We found a parabolic relationship between RUS and FGMs. In other words, FGMs are highest at intermediate levels of urbanization. We suspect the most likely hypothesis to account for this pattern is the presence of ameliorating effects of the environment at either end of our continuum on allostatic load. This idea would suggest that areas of intermediate urbanization are the most stressful (and result in the highest FGMs) and that areas of high and low urbanization are less stressful. Or, as a different perspective of this hypothesis, there are only stress-inducing factors at intermediate levels of urbanization and not at either ends of the continuum.

There is another hypothesis for this parabolic relationship that while we suspect is unlikely, we feel should nonetheless be addressed. Busch and Hayward (2009) describe a parabolic relationship between disturbance and GC levels. At low levels of disturbance, baseline FGMs may be low, but as the disturbance increases, so does the release of GCs. At a point of elevated GCs, the animal enters into a state of chronic stress that eventually becomes pathological, such that with further increased disturbance there is a decrease in baseline plasma GCs or FGMs because of the deactivation of the HPA axis. We feel that Busch and Hayward model does not apply to this system primarily because of evidence of population persistence. Generally, we found higher population densities of marmots in urban areas (rough estimates of up to 50 at the GULS site and down to 10 at the RSP site) suggesting that marmots are doing very well in the urban centers. Additionally, marmots have existed in rural areas for long before the current human population density in the Spokane area, making it unlikely that marmots are now chronically stressed in the rural locations. The important difference between applying the idea that there are ameliorating effects at either end of the continuum idea in our system compared to this idea is that the marmots are not experiencing chronic stress to the degree that the HPA axis is shut down. In other words, the lower levels of FGMs are a result of lower stress.

A suite of environmental factors could account for the ameliorating effects at either end of the continuum, and most likely it is a number of factors acting in conjunction. We suspect that ultimately the parabolic relationship is the result of a trade-offs among environmental factors, such as resource availability and predator pressures, that balance each other in such a way as to result in the parabolic relationship we see between RUS and FGM levels. Even though the young (yearlings and juveniles) and adults exhibit the same parabolic relationship between RUS and FGMs, the vertex of the curve is shifted to the right for adults compared to young, as is shown through the significant interaction effect between RUS and age on FGMs. Therefore, to more specifically explore our hypothesis on why this parabolic relationship may exist, we need to incorporate age into our discussion.

### Interactions between RUS and age on FGM levels along the rural–urban continuum

We found that adults had lower FGM levels in highly urbanized environments and higher FGM levels in more rural environments compared to young marmots. Yearlings and juveniles respond similarly to the same environment. In other words, what may be an ameliorating effect for adults may not be for young. This leaves us with two questions: (i) what specific environmental factors could contribute to the parabolic relationship between RUS and FGMs; and, (ii) why are the vertices of the young and adult curves different? Multiple reasons likely account for both of these, including acclimatization to urban environments, resource availability, predator pressure and population density.

Marmots may be acclimatizing to the presence of humans and human disturbances. While this is not necessarily a universal response, habitation to humans, reflected in behaviors, is found in Olympic marmots (*M. olympus*; Griffin *et al.*, 2007). Habituation to humans, reflected in baseline levels of GCs, has been found in other species including orangutans (*Pongo pygmaeus*; Muehlenbein *et al.*, 2012) and Magellanic penguins (*Spheniscus magellanicus*; Walker *et al.*, 2006). Acclimatization may contribute to why we see lower levels of FGMs in adult marmots in more urban areas. However, we see the opposite pattern in the young, with lower levels of FGMs in the more rural environments. The young may not yet have acclimatized to humans and human disturbances. As a result, the fewer human disturbances they encounter (such as in more rural environments), the lower their allostatic load. Young animals have been shown to be less likely to acclimatize to humans that adults, as found in Magellenic penguins (*Spheniscus magellanicus*; Walker *et al.*, 2005) and white-crowned sparrows (*Zonotrichia leucophrys*; Crino *et al.*, 2011). Walker *et al.* (2005)’s study found that as the chicks got older, their hormonal responses to people changed. However, as juveniles and yearlings in our population exhibit the same FGM suggesting that all we can conclude is that at least through their second summer, their hormonal responses to human disturbances do not change. This does not mean we can claim that acclimatization in not a factor, but rather all we can state is that it may take longer for young marmots to acclimatize than young penguins.

Overall resource availability (such as food and burrow sites) can affect FGMs. Tree lizards (*Urosaurus ornatus*; French *et al.*, 2008) and eastern chipmunks (*Tamias striatus*; Lyons *et al.*, 2017) exhibit lower FGM levels in urban environments and those environments may have more resources available to these organisms. In the Spokane urban area, natural geologic features as well as man-made construction provide an abundance of burrow habitats (pers. obs). Food also varies between urban and rural environments (Lowry *et al.*, 2013 and pers. obs.) and affects levels of glucocorticoids (Busch and Hayward, 2009). Productivity of urban areas can be higher than surrounding natural ones (Shochat *et al.*, 2006). In our urban environments, food is abundant closer to burrows in the urban sites than in the rural ones (pers. obs). Furthermore, the type of food also varies between the environments. The available food at the urban sites is much more influenced by humans. At one site in our study (RLCT), people regularly feed the marmots (e.g. popcorn, nuts, parts of sandwiches). Even at the other urbanized sites, humans influence the diets of the marmot. Manicured lawns provide extensive forage, especially if they contain clover. In contrast, all of the more rural sites (RSP, PNA) have extensive areas of barren ground. In sum, increased resource availability may decrease FGM levels, and it very well may be doing so in adults. But this does not account for young having higher FGM levels in urban areas compared to rural ones.

It should be noted again that we are using FGM levels as a proxy for allostatic load and other factors can affect FGM levels besides allostatic load. For example, Dantzer *et al.* (2011) found that diet can affect FGMs in red squirrels. Squirrels fed conifer seeds had significantly higher FGMs than those fed peanut butter. As we discussed, the diets of the marmots differ along the rural–urban continuum. Therefore, it is possible that the differences we see in FGMs are due to variation in diet and not allostatic load. This is unlikely to be the entire explanation for our results because even if it explains the differences between adults and young, it does not account for the parabolic relationship. Diet is an example of uncontrolled variation that could be affecting our results.

Another factor that can affect allostatic load, and may be most acutely felt by young, is predator pressure. Traditionally, urban environments tend to have fewer predators (McKinney, 2002). Yellow-bellied marmots have predators that likely respond differently to urban pressure. In Colorado, the most prevalent predators of yellow-bellied marmots are coyotes (Van Vuren, 1991, 2001; Armitage, 2014, 1982; Witczuk *et al.*, 2013), badgers (Armitage, 2004), various raptors (Blumstein *et al.*, 2009), weasels, martens, wolves (Frase and Hoffmann, 1980) and black bears (Van Vuren, 2001). Most of the predators listed above are more common in rural environments, but some of them, such as coyotes and raptors, live in urbanized zones, though generally in lower densities. In urban areas, woodchucks are also found in greater abundance in urbanized zones (Lehrer and Schooley, 2010) and experience greater predator pressure in rural environments compared to rural ones (Lehrer *et al.*, 2010). This would suggest that FGM levels of both adults and young should be lower in urban areas. However, not included in the studies listed above are domesticated dogs. The peak in FGM levels in young occurs at a field site adjacent to a dog park. As dogs will prey upon marmots, particularly young ones (pers. obs.), the presence of so many dogs in close proximity to marmot colonies likely contributes to the elevated levels of FGMs in the young. As predation rates are generally higher in young animals than adults (Armitage, 2004; Holmes, 1984), the impact of the proximity of the dog park to the marmot colonies could have a greater effect on young FGM levels than those of adults. While there is not a dog park at the most urban site (RLCT), people walk their dogs there regularly. The frequent exposure to the dogs (via smell, sound,and sight) is likely higher than similar predator exposure in rural environments for young, potentially causing the higher levels of FGMs we see in our more urbanized sites.

Population density has been shown to be positively correlated with glucocorticoids in a diversity of species (Boonstra *et al.*, 1998; Rogovin *et al*., 2003; Cooperman *et al.*, 2004; Kuznetsov *et al*., 2004; Dantzer *et al.*, 2013; Navarro-Castilla *et al.*, 2014). However, we observe the opposite pattern in yellow-bellied marmots: FGMs are lower in urban populations of adults, where density tends to be higher (pers. obs.). This correlation has been found at least in one other species, the prairie vole (*Microtus* ochrogaster; Blondel *et al.*, 2016), but it is much less common than the reverse. Social dynamics associated with population density could be a factor affecting FGM levels (Ebensperger *et al.*, 2011). Creel *et al.* (2013) posit that there are strong interactions between ecological and social factors. Ecological and social factors may act independently, dependently or synergistically on glucocorticoid levels in social species. Possible stability in the social structure, due to greater resource availability in urban environments or social buffering with higher population density (Creel *et al.* 2013), might reduce allostatic load in those environments. Young may be shielded from this competition because they have not reached the age to be included in the social hierarchy (Creel *et al.* 2013). Population density, and its consequences on the social hierarchy, may have a muted effect on the young (as in minimally affect FGM levels) because they do not enter the social hierarchy until their third or fourth year (Armitage, 2014).

**Figure 5:**
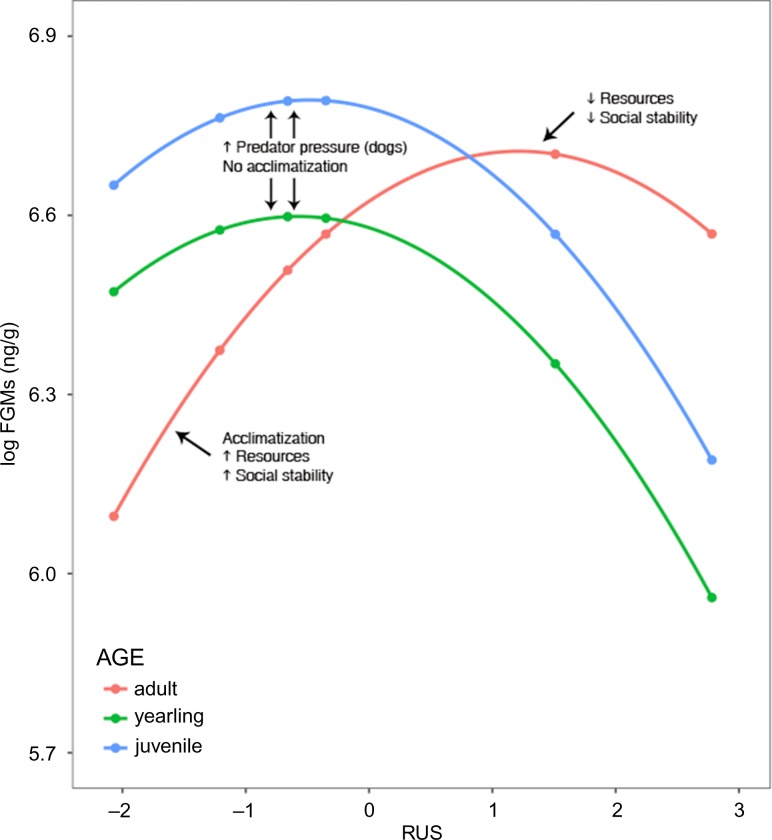
Possible effects of environmental factors contributing to the parabolic relationship between RUS and FGMs.

In conclusion, we found that the relationship between FGM levels and RUS is parabolic for all individuals, but the vertices of those parabolas are different for adults and young. We suspect the most likely explanations for the parabolic relationship between RUS and FGMs are due to acclimatization (or lack of), resource availability, predator pressure and population density. Differences in the location of the vertices of the parabolic relationship between young and adults may be due predator pressure and social dynamics (Fig. 5). Further studies could help clarify the effects of these variables on allostatic load.

The results of this study also suggest that adult yellow-bellied marmots are at the very least urban adaptors but may even be urban exploiters. However, while the young could be urban avoiders based upon their FGM levels, the presence of their parents in urban areas results in their presence also. These results suggest that explicit studies of reproductive success and offspring survival along the urban–rural gradient could provide useful information in helping explain the disparity between adult and young physiological responses to the environment. The results of this study can provide useful information for conservation of less abundant species because it can help us predict how other species may respond. This study also reinforces the importance of examining both adults and young in guiding conservation strategies.

## Supplementary Material

Supplementary Data

Supplementary Data

Supplementary Data

Supplementary Data
