# Supplementary material for: Relaxing life of the city? Allostatic load in yellow-bellied marmots along a rural–urban continuum
Source: Conserv Physiol. 2018 Dec 20;6(1):coy070. doi: 10.1093/conphys/coy070 (PMC6301289; doi:10.1093/conphys/coy070)
Supplement: Supplementary Data [file coy070_som_tables_and_figures_captions.docx]

**SOM Tables and Figures Captions**

SOM Table 1. The location of each field site and each site’s associated RUS. All sites are located in Spokane County, Washington. RUS = rural-urban score.

SOM Table 2. Sample sizes for each age and sex group by trapping site.

SOM Table 3. Means ± SE of each variable included in the PCA.
