# Supplementary material for: Relaxing life of the city? Allostatic load in yellow-bellied marmots along a rural–urban continuum
Source: Conserv Physiol. 2018 Dec 20;6(1):coy070. doi: 10.1093/conphys/coy070 (PMC6301289; doi:10.1093/conphys/coy070)
Supplement: Supplementary Data [file coy070_som_table_1.pdf]

LOCATION	GPS COORDINATES	RUS
Red Lion-Centennial Trail (RLCT)	N47° 39.860' W117° 24.495'	-2.07
Gonzaga University Law School (GULS)	N47° 39.609' W117° 23.892'	-1.21
Witter Aquatic Center (WAC)	N47° 40.199' W117° 23.353'	-0.66
High Bridge Park (HBP)	N47° 39.119' W117° 27.262'	-0.35
Pinecroft Natural Area (PNA)	N47° 40.839' W117° 13.632'	1.51
Riverside State Park (RSP)	N47° 41.743' W117° 29.575'	2.78
