# Supplementary material for: Relaxing life of the city? Allostatic load in yellow-bellied marmots along a rural–urban continuum
Source: Conserv Physiol. 2018 Dec 20;6(1):coy070. doi: 10.1093/conphys/coy070 (PMC6301289; doi:10.1093/conphys/coy070)
Supplement: Supplementary Data [file coy070_som_table_2.pdf]

| | RLCT (-2.07) | GULS (-1.21) | WAC (-0.66) | HBP (-0.35) | PNA (1.51) | RSP (2.78) |
|---|---|---|---|---|---|---|
| Adult F | 2 | 9 | 2 | 10 | 7 | 1 |
| Adult M | 1 | 8 | 1 | 3 | 5 | 0 |
| Yearling F | 0 | 5 | 0 | 1 | 3 | 1 |
| Yearling M | 1 | 3 | 1 | 1 | 1 | 0 |
| Juvenile F | 1 | 4 | 5 | 1 | 2 | 2 |
| Juvenile M | 3 | 6 | 4 | 1 | 1 | 2 |
