# Supplementary material for: Relaxing life of the city? Allostatic load in yellow-bellied marmots along a rural–urban continuum
Source: Conserv Physiol. 2018 Dec 20;6(1):coy070. doi: 10.1093/conphys/coy070 (PMC6301289; doi:10.1093/conphys/coy070)
Supplement: Supplementary Data [file coy070_som_table_3.pdf]

VARIABLE	MEAN ± SE
Developed-open space	0.113 ± 0.05
Developed-low intensity	0.333 ± 0.13
Developed-medium intensity	0.098 ± 0.06
Developed-high intensity	0.034 ± 0.02
Evergreen forest	0.230 ± 0.10
Shrub/scrub	0.120 ± 0.08
Grassland herbaceous	0.005 ± 0.005
People/ dogs/ cars/ bikes (number of individuals)	29.5 ± 14.26
Sound intensity (decibels)	69.551 ± 1.02
